# Effects of Proton Pump Inhibitor Therapy, *H. pylori* Infection and Gastric Preneoplastic Pathology on Fasting Serum Gastrin Concentrations

**DOI:** 10.3389/fendo.2021.741887

**Published:** 2021-11-18

**Authors:** Reuben Veysey-Smith, Andrew R. Moore, Senthil V. Murugesan, Laszlo Tiszlavicz, Graham J. Dockray, Andrea Varro, D. Mark Pritchard

**Affiliations:** ^1^ Institute of Systems, Molecular and Integrative Biology, University of Liverpool, Liverpool, United Kingdom; ^2^ Gastroenterology Department, Liverpool University Hospitals NHS Foundation Trust, Liverpool, United Kingdom; ^3^ Gastroenterology Department, Blackpool Teaching Hospitals NHS Foundation Trust, Blackpool, United Kingdom; ^4^ Department of Pathology, University of Szeged, Szeged, Hungary

**Keywords:** gastrin, proton pump inhibitor, *Helicobacter pylori*, atrophic gastritis, oesophagogastroduodenoscopy

## Abstract

**Background:**

Hypergastrinaemia occasionally indicates the presence of a gastrinoma. However it is much more commonly associated with various benign causes including proton pump inhibitor (PPI) use, *Helicobacter pylori* infection and/or atrophic gastritis. The extent to which these factors interact to influence fasting serum gastrin concentrations remains incompletely understood.

**Materials and Methods:**

Fasting serum gastrin concentrations were measured by radioimmunoassay in 1,400 patients attending for diagnostic oesophagogastro-duodenoscopy. After exclusions, 982 patients were divided into four groups and their results analysed. We compared gastrin concentrations in normal patients (no *H. pylori* infection, no PPI use and no histological evidence of gastric preneoplasia (n=233)), with those in patients who were taking regular PPIs (*H. pylori* negative with no gastric preneoplasia (n=301)), patients who had active *H. pylori* infection but no gastric preneoplasia (n=164) and patients with histologically confirmed gastric preneoplasia (n=284).

**Results:**

Median fasting gastrin concentration in the normal group was 20pM and was significantly increased in PPI users (46pM, p<0.0001), patients with active *H. pylori* infection (27pM, p<0.0001), and patients with antral (25pM, p<0.01) or corpus (48pM, p<0.0001) gastric preneoplasia. PPI use resulted in further significant increases in fasting serum gastrin concentrations in patients who were infected with *H. pylori* (50pM, n=56) or who had antral gastric preneoplasia (53pM, n=87), but did not significantly alter serum gastrin concentrations in patients with corpus preneoplasia (90pM, n=66).

**Conclusions:**

PPI use, *H. pylori* infection and atrophic gastritis all caused significant elevations of median fasting gastrin concentrations. However, several patients who had potential risk factors for hypergastrinaemia still demonstrated fasting serum gastrin concentrations within the normal range.

## Introduction

Gastrin is the major gastric hormone responsible for regulating gastric acid secretion and growth of the oxyntic mucosa (1). Pyloric antral mucosal G-cells synthesise gastrin as a 101-amino acid precursor (preprogastrin) that is processed to yield biologically active amidated gastrin-17 and gastrin-34 (2, 3). These amidated forms of gastrin bind to cholecystokinin (CCK)-2 receptors on gastric enterochromaffin-like (ECL)-cells, stimulating the release of histamine which acts in a paracrine manner, binding to histamine H2-receptors on parietal cells and resulting in acid secretion.

Increased fasting serum gastrin concentrations are encountered in several clinical conditions. Arguably the most serious of these is a gastrin-secreting tumour (gastrinoma) leading to the hypersecretion of gastric acid and the clinical manifestations of Zollinger-Ellison Syndrome (ZES). Although some patients with ZES have very high fasting serum gastrin concentrations, this is by no means universal and the magnitude of hypergastrinaemia is not a reliable indicator of its cause. A number of other clinical conditions can also lead to elevated fasting serum gastrin concentrations. These include atrophic gastritis, either caused by autoimmune disease or chronic *H. pylori* infection, which results in hypochlorhydria and compensatory hypergastrinaemia. Hypochlorhydria can also result from pharmacological inhibition of gastric acid secretion by proton pump inhibitor (PPI) and to a lesser extent H2-receptor antagonist drugs. The resulting increase in fasting gastrin concentration is only usually mild-moderate (4) with values usually less than three times the upper limit of normal (5). However, there have been studies suggesting a marked increase in fasting gastrin concentrations in some patients on PPI therapy (6–8). However, in these studies, patients with confounding factors, such as active *H. pylori* infection and/or the presence of atrophic gastritis were not excluded, thus making it difficult to determine whether these confounding factors or PPI usage primarily drive increased gastrin concentrations. Finally, antral predominant *H. pylori-*associated gastritis has also been reported to locally decrease the expression of the gastrin-inhibiting peptide somatostatin (9). However, the resultant increase in gastrin secretion in this scenario is usually only mild.

The hypergastrinaemia associated with these various benign causes may itself have biological and clinical consequences. For example, there is evidence, at least from animal models, that gastrin acts as a cofactor during *H. pylori* associated gastric carcinogenesis and the hypergastrinaemia associated with autoimmune atrophic gastritis is also crucial for the development of type 1 gastric neuroendocrine tumours (10–12). PPI-induced hypergastrinaemia has also been proposed to promote the neoplastic progression of Barrett’s oesophagus in some patients (13, 14).

As there is considerable overlap between the fasting serum gastrin concentrations observed in patients with ZES and patients who have atrophic gastritis, PPI use or *H. pylori* infection (5), it would clinically helpful to understand how these various factors singly and in combination influence fasting serum gastrin concentrations in routine clinical practice. In addition there is increasing concern about the possibility that hypergastrinaemia resulting from PPI use may be a contributory factor in the pathogenesis of gastric and oesophageal cancers in some patients. We therefore set out to understand how these factors influenced fasting serum gastrin concentrations in a large cohort of patients attending for elective outpatient diagnostic oesophagogastroduodenoscopy.

## Methods

### Ethical Approval and Funding

This study was funded by the National Institute for Health Research (NIHR) *via* the Liverpool Biomedical Research Centre (BRC) and was sponsored by Royal Liverpool and Broadgreen University Hospitals NHS trust (R&D No. 3592) and the University of Liverpool. Local ethics committee (Liverpool [Adult] Research Ethics Committee REC:08/H1005/37) approval was obtained (15).

### Study Design and Population

Participants were recruited between May 2008 and July 2011 and provided informed consent. All participants attended the Gastroenterology Unit of Royal Liverpool University Hospital for an elective outpatient upper gastrointestinal (GI) endoscopy, mostly to investigate upper gastrointestinal symptoms or anaemia. Patients who were attending for emergency endoscopy, surveillance of a known condition such as Barrett’s oesophagus or planned endoscopic therapy were not included. Inclusion criteria were age 18 years or older, capable of giving informed written consent and had a clinical indication for elective diagnostic (non-surveillance or therapeutic) upper GI endoscopy. Exclusion criteria were haemodynamic instability or active bleeding at endoscopy; moribund or established terminal malignancy; hepatic cirrhosis; bleeding diathesis or current anticoagulation; pregnancy; HIV, hepatitis B or hepatitis C infection or other contraindication to endoscopy. Full demographic information, past medical history, concurrent medical treatment and the results of previous *H. pylori* testing, or treatment were recorded. We did not keep a formal record of patients who declined to participate in the study, but estimate that this was only approximately 5% of those who were approached.

Patients fasted for a minimum of 12 hours before the procedure. During the endoscopy, all macroscopic abnormalities were recorded and any clinically indicated biopsies were performed. In addition, biopsies of the antrum and corpus (minimum 2 per site) were taken for histological analysis and an additional two gastric biopsies were taken for a rapid urease test (*Pronto Dry*, MIC, Brignais, France). Before the procedure, patients had blood drawn for analysis of fasting serum gastrin concentration and *H. pylori* serology (by IgG ELISA, Biohit, Barcelona, Spain).

### Gastrin Analysis

Fasting serum gastrin concentrations were measured using a well validated radioimmunoassay (RIA) using rabbit polyclonal antibody L2 that reacts equally with G17 and G34, but not with biologically inactive C-terminal extensions or deletions of these peptides (16).

### Histopathology

Histopathological specimens were stained with haematoxylin and eosin and any special stains at the discretion of the reporting histopathologist. The samples were initially reported by one of the hospital’s specialist gastrointestinal histopathologists as part of routine clinical care. The samples were subsequently reviewed by a single expert external gastrointestinal pathologist (LT). If either of these pathologists reported the presence of atrophic gastritis, intestinal metaplasia or dysplasia in the antrum and/or corpus, this was considered to represent preneoplasia at that anatomical site.

### Patient Groups

**Figure 1:**
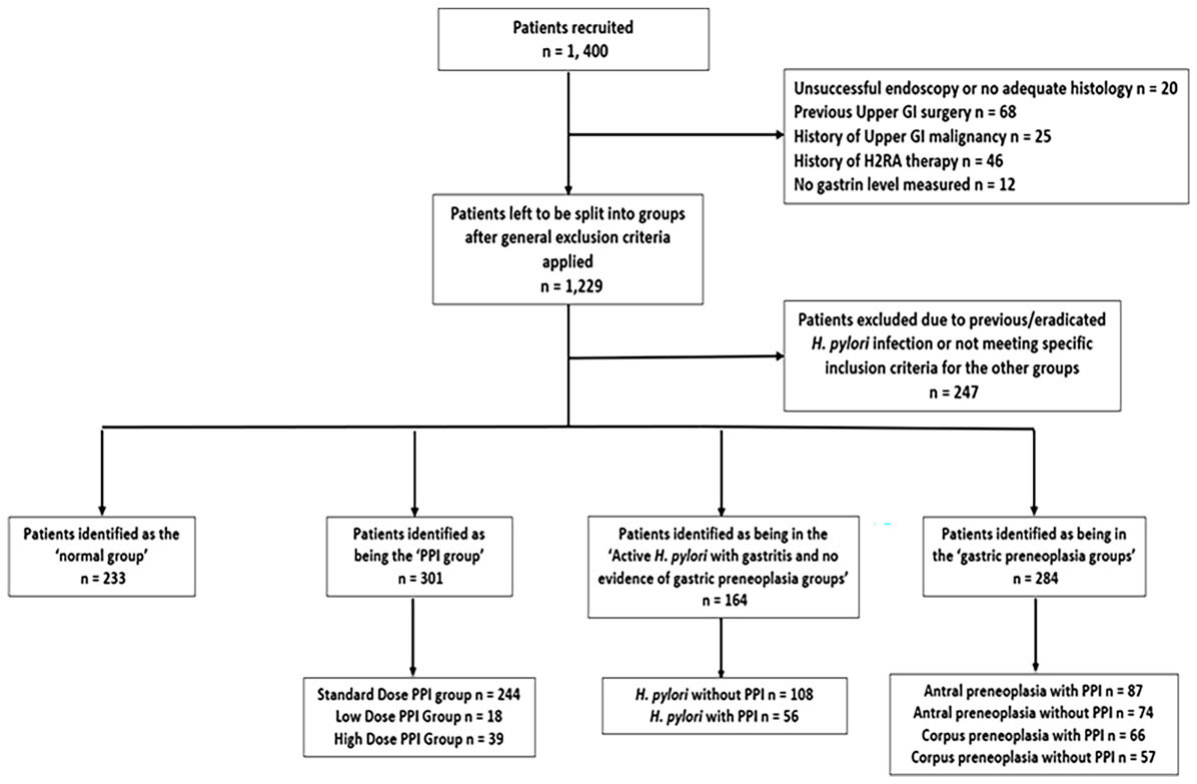
Flowchart demonstrating how participants recruited from the database were excluded and then assigned into groups.

Patients were divided into four groups on the basis of criteria as shown in **Figure 1**. Some patients (n=171) were initially excluded because incomplete biopsy or blood samples were obtained or because they had confirmed upper GI malignancy, had undergone previous oesophagogastric surgery or were taking H2 receptor antagonists. An additional 247 patients were excluded because they had positive or indeterminate *H. pylori* serology suggesting previous *H. pylori* infection, but they had no current evidence of active infection by histology or rapid urease test and did not have histological evidence of gastric preneoplastic pathology, so they could not be accurately attributed to one of the analysis groups. The remaining 982 patients were then divided into 4 groups and included in the analyses. Patients in the ‘normal’ group (n=233) had no evidence of gastric or duodenal ulceration or malignancy (but could have other minor benign findings such as gastritis, duodenitis, oesophagitis or benign gastric fundic cystic gland polyps), were not taking any PPIs within the last 2 weeks, had no evidence of previous or active *H. pylori* infection (as demonstrated by negative gastric histology, rapid urease test and serology), and had no evidence of gastric malignant or premalignant histology. Patients in the ‘PPI user’ group (n=301) were taking a PPI drug regularly (i.e. not on an ‘as required’ basis) with the last dose being taken within two weeks of the endoscopy procedure, had no evidence of previous or current *H. pylori* infection by the same criteria as mentioned above and had no evidence of gastric malignant or premalignant pathology. This group was then further divided into standard (n=244), high (n=39) and low dose (n=18) PPI groups based on the British National Formulary (BNF) guidance for PPI prescribing (17) (e.g. for omeprazole, doses <20mg daily, 20mg daily and >20mg daily were considered as low, standard and high doses). Patients in the active *H. pylori* infection group (n=164) had evidence of active *H. pylori* infection, defined by either a positive histology result or by both positive *H. pylori* serology and rapid urease test results if histology was negative. Patients who had any histopathological evidence of gastric preneoplasia were excluded from this group. This group was divided further into those taking PPI therapy within the last 2 weeks (n=56) and those not (n=108). Patients in the gastric preneoplasia group (n=284) had gastric atrophy/IM/dysplasia reported by at least one of the two independent histopathologists. This group was divided into patients who showed preneoplasia in the antrum only (n=161) and those who showed preneoplasia in the corpus (potentially in addition to the antrum) (n=123) and these groups were also considered separately depending on whether they were taking PPIs within the last 2 weeks or not (see **Figure 1**).

### Statistics and Analysis

All statistical analysis was carried out on IBM SPSS Statistics 26. Groups were compared using an unpaired Mann-Whitney U and Kruskal Wallis tests. Due to multiple testing, a Bonferroni adjustment was carried out on each p-value obtained. A Pearson’s correlation coefficient was used to analyse the influence of age on fasting serum gastrin concentration in the normal patient group. All graphs were created on GraphPad Prism 9.0.0. Outliers were defined in each group by the Tukey method (Q3 + 1.5IQR).

## Results

### Patient Characteristics

The 1,400 patients recruited in this study had the following characteristics (as previously described) (15): females 57.5%, median age 60 years (interquartile range of 48-70 years) and 98.4% were Caucasian. More than half the total cohort (52.3%) reported PPI use, 21.8% were *H. pylori* positive by histology, and 43.3% were positive by IgG serology. Within the total cohort, we detected 21 cancers (12 gastric, 9 oesophageal), 54 peptic ulcers and 67 cases of Barrett’s oesophagus. No patients were diagnosed as having a gastrinoma, Zollinger Ellison syndrome or multiple endocrine neoplasia (MEN)-1. 443 patients were reported as having no macroscopic upper gastrointestinal abnormality and the other patients had various benign pathologies such as oesophagitis, gastritis, duodenitis, benign polyps or vascular lesions.

After excluding 418 patients for various reasons as shown in **Figure 1**, the remaining 982 patients were subdivided into four groups as described above: normal patients [no *H. pylori* infection, no PPI use and no histological evidence of gastric preneoplasia (n=233)]; patients who were taking regular PPIs [*H. pylori* negative with no gastric preneoplasia (n=301)]; patients who had active *H. pylori* infection but no gastric preneoplasia (n=164) and patients with histologically confirmed gastric preneoplasia (n=284). There was a significant difference between the age profiles of these four main groups (Kruskal Wallis p<0.001). Specifically, the patients who had gastric preneoplasia were significantly older than those in the other three groups (p<0.0001 by Mann Whitney U test for each group compared to control). This difference was biologically plausible. However, within the normal patient group, there was no significant correlation between fasting serum gastrin concentration and age (Pearson’s correlation coefficient = 0.0661).

### Fasting Serum Gastrin Concentrations in the Normal Patient Group

**Figure 2:**
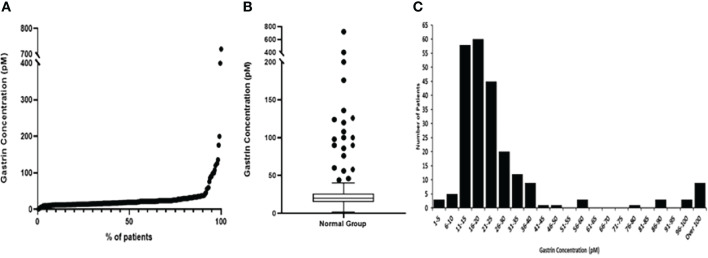
Graphs representing fasting serum gastrin concentrations (pM) in the normal group of patients. **(A)** Scatter graph showing the fasting serum gastrin concentrations (pM) from minimum to maximum values **(B)** Box and Whisker plot, showing the median gastrin concentration, plotted using the Tukey method **(C)** Histogram showing the spread of results.

The median fasting gastrin concentration for the normal patient group was 20pM. However, as demonstrated in **Figure 2** and **Table 1**, although most patients had low concentrations (IQR 15-26pM), the full range of concentrations was much larger (1-720pM).

**Table 1:** Median fasting gastrin concentrations from each group included in this study.

	Normal Group	PPI groups				Active *H. pylori* with gastritis groups			Preneoplasia groups				
		All PPI	Standard Dose	High Dose	Low dose	All *H. pylori*	*H. pylori* without PPI	*H. pylori* with PPI	All preneoplasia patients	Antral preneoplasia with PPI	Antral preneoplasia without PPI	Corpus preneoplasia with PPI	Corpus preneoplasia without PPI
**Number of Patients**	233	301	244	39	18	164	108	56	284	87	74	66	57
**Median age (IQR)**	52 (41-65)	57 (47-67)	58 (48-67)	55 (44-65)	62 (52-67)	56 (46-66)	56 (46-67)	59 (45-64)	65 (53-74)	64 (53-72)	65 (52-73)	70 (61-77)	64 (49-74)
**Age range**	18-93	17-91	17-91	26-88	23-79	22-86	22-86	23-83	21-88	21-86	24-87	23-88	27-86
**Minimum value**	1	9	9	11	11	3	3	8	10	11	10	10	11
**Q1**	15	29	30	30	25	22	20	33	24	32	19	38	26
**Median**	20	46	46	72	31	32	27	50	45	53	25	90	48
**Q3**	26	84	80	131	52	62	48	109	116	100	42	186	260
**Maximum value**	720	650	440	650	128	295	295	295	1867	545	380	1290	1867
**Inter-quartile range (IQR)**	11	56	50	101	27	40	29	76	92	69	23	148	234
**Number >40pM**	21 (9%)	168 (56%)	138 (57%)	25 (64%)	5 (28%)	64 (39%)	32 (30%)	32 (57%)	152 (54%)	51 (59%)	22 (30%)	48 (73%)	31 (54%)
**Number >100pM**	9 (4%)	52 (17%)	37 (15%)	12 (31%)	3 (17%)	24 (15%)	7 (6%)	17 (30%)	79 (28%)	22 (25%)	8 (11%)	29 (44%)	20 (35%)

We have previously analysed the fasting gastrin concentrations in a subset of 126 of these patients using more stringent criteria for categorising normality (in particular this smaller group excluded patients who had minor benign endoscopic abnormalities such as gastritis or duodenitis and patients who were taking NSAIDs or aspirin). That analysis demonstrated that the upper limit of the normal range for this assay was 40pM, but 5 of these 126 apparently normal patients (4%) still showed elevated gastrin concentrations.

In the current analysis, 21 (9.0%) of the 233 patients in the normal group had fasting serum gastrin concentrations >40pM (the defined upper limit of normal for this assay) of whom 9 (3.8%) had concentrations >100pM (2.5 times the upper limit of normal for the assay). Further review of all 21 individual case records demonstrated that 6 of these patients (including 4 who had fasting serum gastrin concentrations >100pM) had significant renal impairment (CKD 3 or 4) and this was the likely explanation for their hypergastrinaemia. None of the remaining 15 patients were diagnosed as having a gastrinoma, multiple endocrine neoplasia-1 or pernicious anaemia in the 10 years since the original analysis was undertaken, although some patients did not undergo follow up for that entire period at our centre. No apparent cause for fasting serum gastrin concentrations above the accepted normal range (>40pM) was therefore demonstrated in 6.4% of apparently normal subjects.

### Comparison of Fasting Serum Gastrin Concentrations Between All Patient Groups

Fasting serum gastrin concentrations were significantly different between the normal patient group, the patients receiving PPIs, the patients infected with *H. pylori* (but who were not taking PPIs) and the patients with gastric preneoplasia (but who were not taking PPIs) (p<0.001 Kruskal Wallis).

### Effects of PPI Use on Fasting Serum Gastrin Concentrations

**Figure 3:**
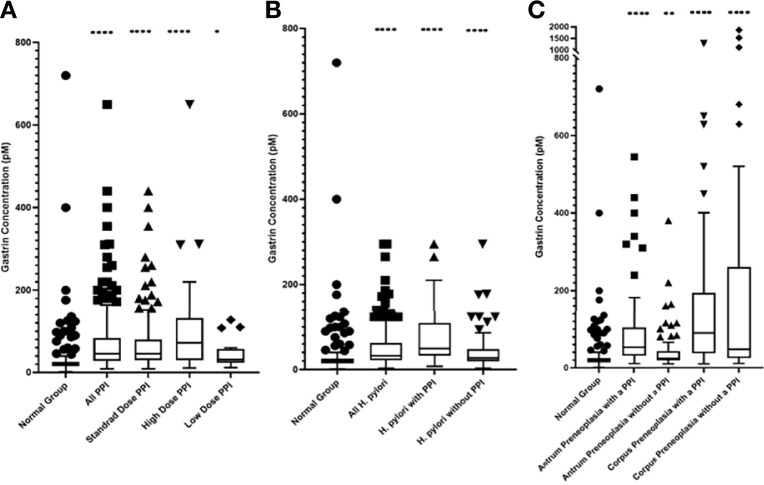
Box and Whisker plots, plotted using the Tukey method, showing the median fasting serum gastrin concentrations in patients within each group **(A)** PPI treated groups **(B)** Active *H. pylori* with gastritis but no evidence of gastric preneoplasia (atrophy/IM) groups **(C)** Gastric preneoplasia (atrophy/IM) groups. ****p < 0.0001, **p < 0.01, *p < 0.05 compared against the normal group (Mann-Whitney U test).

**Figure 4:**
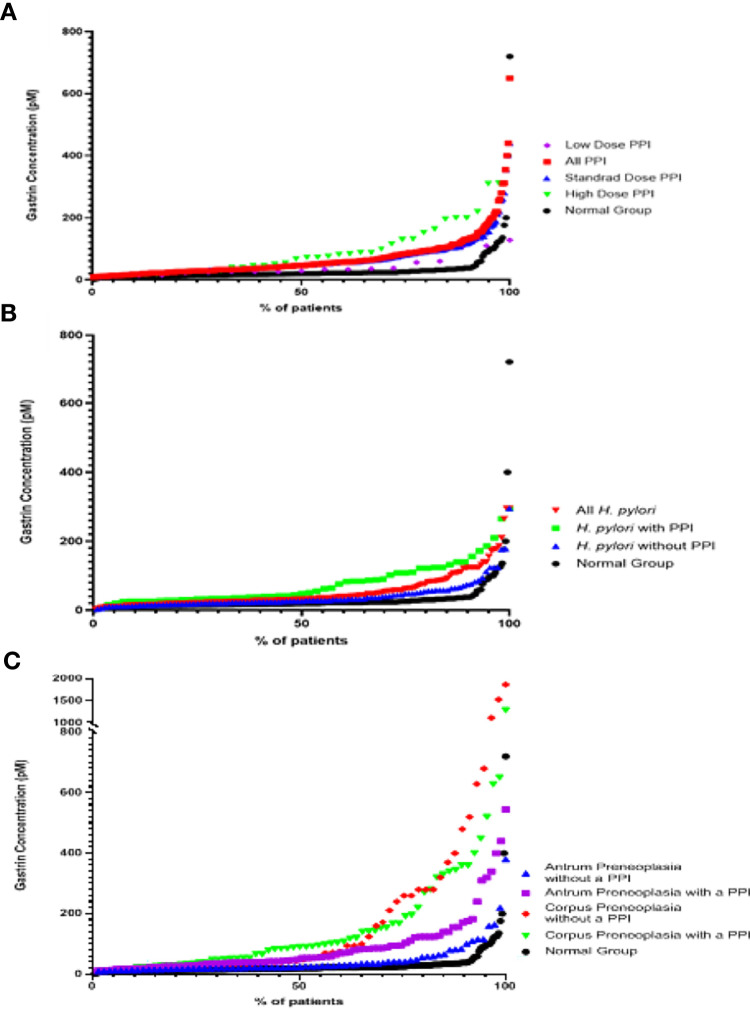
Scatter graphs showing fasting serum gastrin concentrations in patients from the different groups **(A)** PPI groups **(B)** Active *H. pylori* with gastritis but no evidence of gastric preneoplasia (atrophy/IM) groups **(C)** Gastric preneoplasia (Atrophy/IM) groups.

As shown in **Figures 3** and **4** and **Tables 1** and **2**, the ‘PPI user’ group had significantly higher fasting serum gastrin concentrations (median 46pM, IQR 29-84pM) than the normal patient group (median 20pM, IQR 15-26pM, p < 0.0001 Mann Whitney U test). All three PPI dose groups also had significantly higher gastrin concentrations than the normal group: standard dose PPI users (median 46pM, IQR 30-80pM, p < 0.0001 Mann Whitney U test), high-dose PPI users (median 72pM, IQR 30-131pM, p < 0.0001 Mann Whitney U test) and low-dose PPI users (median 31pM, IQR 25-52pM, p < 0.05 Mann Whitney U test). However, there were however no significant differences in fasting serum gastrin concentrations between patients taking standard, low and high doses of PPI (Kruskal Wallis test). However, the sample sizes for the low and high dose PPI groups (n = 39 and n = 18 respectively) were much lower than standard dose group (n = 244), potentially influencing the results of this statistical analysis.

**Table 2:** Mann-Whitney U tests comparing the median fasting gastrin concentrations between groups.

Group comparison		Median	Difference between medians		p-value	Adjusted p value~
Group 1	Group 2	Group 1: Group 2	Actual difference	Hodges-Lehmann adjusted difference		
Normal group	All PPI users	20: 46	26	24	**<0.0001**	**<0.0001**
	Standard dose PPI users	20: 46	26	24	**<0.0001**	**<0.0001**
	High dose PPI users	20: 72	52	42	**<0.0001**	**<0.0001**
	Low dose PPI users	20: 31	11	11	**0.0012**	**0.0300**
	All active *H. pylori*	20: 32	12	12	**<0.0001**	**<0.0001**
	Active *H. pylori* with PPI	20: 50	30	27	**<0.0001**	**<0.0001**
	Active *H. pylori* without PPI	20: 27	7	7	**<0.0001**	**<0.0001**
	Antrum preneoplasia with PPI	20: 53	33	28	**<0.0001**	**<0.0001**
	Antrum preneoplasia without PPI	20: 25	5	6	**<0.0001**	**0.0018**
	Corpus preneoplasia with PPI	20: 90	70	68	**<0.0001**	**<0.0001**
	Corpus preneoplasia without PPI	20: 48	28	27	**<0.0001**	**<0.0001**
All PPI users	Active *H. pylori* without PPI	46: 27	19	16	**<0.0001**	**<0.0001**
	Active *H. pylori* with PPI	46: 50	4	7	0.1273	>0.999
	Antrum preneoplasia with a PPI	46: 53	7	6	0.1568	>0.999
	Antrum preneoplasia without a PPI	46: 25	21	16	**<0.0001**	**<0.0001**
	Corpus preneoplasia with a PPI	46: 90	44	36	**<0.0001**	**0.0007**
	Corpus preneoplasia without a PPI	46: 48	2	8	0.1765	>0.999
Standard dose PPI users	High dose PPI users	46: 72	26	14	0.0755	>0.999
	Low dose PPI users	46: 31	15	12	0.0669	>0.999
High dose PPI users	Low dose PPI users	72: 31	41	26	0.0197	0.4925
*H. pylori* with PPI	*H. pylori* without PPI	50: 27	23	21	**<0.0001**	**<0.0001**
Antrum preneoplasia with PPI	Antrum preneoplasia without PPI	53: 25	28	20	**<0.0001**	**<0.0001**
	Corpus preneoplasia with PPI	53: 90	37	26	0.0129	0.0645
Corpus preneoplasia without PPI	Corpus preneoplasia with PPI	48: 90	42	13	0.2617	>0.999
	Antrum preneoplasia without PPI	48: 25	23	20	**0.0002**	**0.005**

~p values were adjusted using the Bonferroni correction for multiple comparisons.

Bold values are statistically significant.

Interestingly 133 (44%) of 301 PPI treated patients still had fasting serum gastrin concentrations within the normal range (<40pM). 52 (17%) of these PPI treated patients had marked hypergastrinaemia (defined as fasting gastrin >100pM or 2.5 times the upper of limit of normal).

### Effects of Active *H. pylori* Infection on Fasting Serum Gastrin Concentrations

Fasting serum gastrin concentrations in patients who had active *H. pylori* infection (but no gastric preneoplasia or PPI use) were slightly, but significantly higher than those found in the normal patient group (median 27pM IQR 20-48pM vs 20pM IQR 15-26pM, p < 0.0001 Mann Whitney U test) (**Figures 3**, **4** and **Table 1**).

76 (70%) of 108 *H. pylori* infected patients who were not taking a PPI had fasting serum gastrin concentrations within the normal range (<40pM). Only 7 (6%) *H. pylori* infected patients in this group had evidence of marked hypergastrinaemia (>100pM).

### Effects of Concurrent PPI Usage in Patients Who Also Had Active *H.* pylori Infection

Patients who had active *H. pylori* infection and who were also taking PPIs had significantly higher fasting serum gastrin concentrations (median 50pM, IQR 33-109pM) than those who had active *H. pylori* alone (median 27pM IQR 20-48pM, p<0.0001 Mann Whitney U test). There was however no significant difference in fasting gastrin concentrations between *H. pylori* infected patients who were taking PPIs and uninfected patients who were taking PPIs (median 50pM IQR 33-109pM v 46pM IQR 29-84pM, p=0.13 Mann Whitney U test).

### Effects of Gastric Preneoplasia on Fasting Serum Gastrin Concentrations

Patients who had preneoplastic changes in at least the gastric corpus had significantly higher fasting serum gastrin concentrations (median 48pM IQR 26-260pM, p < 0.0001 Mann Whitney U test) than the normal group (median 20pM, IQR 15-26pM). Patients who had corpus or pangastric preneoplastic changes also had significantly higher fasting serum gastrin concentrations than the patients who solely had antral preneoplasia (median 48pM IQR 26-260pM v 25pM, IQR 19-42pM, p < 0.01 Mann Whitney U test). Patients with antral preneoplasia who were not taking PPIs, showed minimally elevated fasting gastrin concentrations compared to the normal patient group (median 25pM IQR 19-42pM v 20pM IQR 15-26pM), but this was still statistically significant (p < 0.01 Mann Whitney U test) (**Figures 3**, **4** and **Table 1**).

26 (46%) of 57 patients with corpus preneoplasia who were not taking PPIs nevertheless exhibited fasting serum gastrin concentrations within the normal range (<40pM) and 20 (35%) exhibited marked hypergastrinaemia (>100pM). 52 (70%) of 74 patients with antral preneoplasia who were not taking PPIs nevertheless exhibited fasting serum gastrin concentrations within the normal range (<40pM) but only 8 (11%) were markedly hypergastrinaemic (>100pM).

### Effects of Concurrent PPI Usage in Patients Who Also Had Gastric Preneoplasia

Concurrent PPI use resulted in significantly higher fasting serum gastrin concentrations in patients with antral preneoplasia (median 53pM IQR 32-100pM) compared to patients with the same pathology who were not taking PPIs (median 25pM IQR 19-42pM, p < 0.0001 Mann Whitney U test) and normal control subjects (median 20pM, IQR 15-26pM, p < 0.0001 Mann Whitney U test). However, the fasting serum gastrin concentrations in PPI treated patients who had antral preneoplasia were not significantly different from those observed in PPI-treated patients who had no evidence of either gastric preneoplasia or *H. pylori* infection (median 53pM IQR 32-100pM v 46pM IQR 30-80pM, p = 0.16 Mann Whitney U test).

Fasting serum gastrin concentrations in patients who had corpus preneoplasia, but who were not taking a PPI were not significantly different from those found in PPI treated patients with no gastric preneoplasia (median 48pM IQR 26-260pM v 46pM IQR 29-84pM, p = 0.18 Mann Whitney U test). However, patients who had corpus preneoplasia and who were also taking PPIs had significantly higher fasting serum gastrin concentrations compared to non-preneoplastic patients who were taking PPIs (median 90pM IQR 38-186pM v 46pM IQR 30-80pM, p < 0.0001 Mann Whitney U test) and antral preneoplasia patients who were taking PPIs (median 90pM IQR 38-186pM vs 53pM IQR 32-100pM, p < 0.0001 Mann Whitney U test). Interestingly, although the median fasting serum gastrin concentration in PPI-treated corpus preneoplasia patients was higher than that observed in corpus preneoplasia patients who were not taking PPIs (90pM IQR 38-186pM v 48pM IQR 26-260pM), this difference was not statistically significant (p = 0.26 Mann Whitney U test).

## Discussion

This study has analysed the contribution of several factors which are known to influence fasting serum gastrin concentrations. We studied a large cohort of patients who underwent elective diagnostic oesophagogastroduodenoscopy as part of their routine clinical investigative pathway. The results demonstrated that although the majority of normal subjects (91%) had fasting serum gastrin concentrations within the normal range (<40pM), a significant minority (6.4%) had elevated concentrations without an apparent cause. PPI use, *H. pylori* infection and gastric preneoplastic pathology all individually caused significant elevations of fasting serum gastrin concentrations, with corpus preneoplasia appearing to cause the most marked effect. PPI use resulted in further elevations of fasting serum gastrin concentrations in patients who had *H. pylori* infection or histological gastric antral preneoplasia, but the magnitude of the increase was similar to that which was observed in otherwise normal subjects. This suggests that PPI use induces similar degrees of hypergastrinaemia patients who also have other risk factors for increased gastrin production.

We initially examined fasting serum gastrin concentrations in patients who had no known risk factors for hypergastrinaemia (gastrinoma, PPI use, *H. pylori* infection or atrophic gastritis). Most of these subjects (>90%) had fasting serum gastrin concentrations below the upper limit of the normal range (40pM), but a significant minority had elevated concentrations. Some of these cases were attributed to chronic kidney disease, which is known to influence gastrin concentrations because gastrin is partially cleared from circulation by renal metabolism (5). Explanations for hypergastrinaemia were however not found in 15 patients. Potential reasons could include undiagnosed atrophic gastritis (due to biopsy sampling), patient misreporting of acid suppressing drug usage or incomplete fasting. Spurious assay readings may have also contributed in some cases, but unfortunately repeat blood samples were not routinely analysed within this study. Nonetheless, it appears that a small proportion of normal people have fasting serum gastrin concentrations above the upper limit of normal without having an obvious explanation. This should be born in mind when trying to investigate the cause of hypergastrinaemia in an individual patient, as occasionally no explanation may be found and in the absence of a suggestive clinical history, exhaustive additional investigations for a gastrinoma may not always be indicated.

As demonstrated in many previous studies (18), PPI use resulted in hypergastrinaemia by inhibiting gastric acid secretion (**Figures 3**, **4**). Although in some studies PPI use has been found to markedly increase gastrin concentrations (6, 7, 19), the extent of the increase found in this study was moderate in most patients. The strength of the present study however is that it removed confounders that may have increased fasting serum gastrin concentrations, such as active *H. pylori* infection and atrophic gastritis and it also used a well validated radioimmunoassay. Previous studies have reported marked variability between different commercial gastrin assays (20). Median fasting serum gastrin concentrations were approximately 2 fold higher in PPI treated patients. However, 44% of PPI patients still had fasting serum gastrin concentrations within the normal range and only 17% had concentrations >100pM, which are the concentrations that are potentially more likely to have biological consequences. It therefore appears that PPI does not induce hypergastrinaemia universally in all patients who take this medication and very high concentrations only appear to occur in a small proportion of subjects.

We also attempted to investigate the relationship between PPI dose and fasting serum gastrin concentrations. Although previous studies have suggested that increased PPI doses are more likely to be associated with increased gastrin concentrations (21), our results showed a trend but no statistically significant association. However there were few patients in the high and low dose PPI groups (n = 39, n = 18 respectively), compared to those taking a standard dose (n = 244), so this is likely to have influenced the analysis.

Active *H. pylori* infection is also known to cause hypergastrinaemia in some subjects (5). Fasting serum gastrin concentrations were significantly elevated in patients who had active *H. pylori* infection, but no other apparent cause of hypergastrinaemia. However, the magnitude of the effect was very small and 70% of patients in this group still had fasting serum gastrin concentrations within the normal range. This is in keeping with previous work which has suggested that *H. pylori* gastritis without atrophy/IM only has mild effects on serum gastrin concentrations as a result of decreased local somatostatin expression in patients with antral-predominant gastritis (9) or decreased acid secretion in patients with corpus gastritis (22). PPI use resulted in a further elevation of fasting serum gastrin concentrations in *H. pylori* infected patients, but the degree of elevation was not significantly different from that observed in PPI treated uninfected patients. This suggests that PPI use has a larger impact on gastrin production than that induced by active *H. pylori* infection in the absence of gastric mucosal atrophy.

Chronic atrophic corpus gastritis elevates fasting serum gastrin concentrations by destruction of the oxyntic mucosa, leading to hypochlorhydria and unopposed gastrin secretion (23). Patients in this study who had corpus preneoplastic pathology had significantly elevated fasting serum gastrin concentrations compared to the normal group. However, 46% of patients still had concentrations within the normal range, suggesting that adequate acid secretion is preserved in a proportion of these subjects, in keeping with the multi-focal nature of *H. pylori* induced atrophic gastritis in some patients. The degree of hypergastrinaemia was much smaller in the antral-predominant gastric preneoplasia group (with 70% of patients still having fasting serum gastrin concentrations within the normal range) and in this case it is probably due to decreased secretion of somatostatin rather than a consequence of decreased acid secretion (9, 23). This is in keeping with the further increase in fasting serum gastrin concentrations that were observed in PPI treated antral preneoplasia patients, where hypochlorhydria was likely to be the causative mechanism. By contrast, PPI use did not result in a significant additional increase in fasting serum gastrin concentrations in patients who also had pre-existing corpus preneoplasia. This is not necessarily surprising as many of those patients will already have reduced acid secretion as a result of atrophic gastritis. An additive effect may have occurred in those patients who had incomplete atrophic gastritis and some residual gastric acid secretion, but our study was probably underpowered to detect this.

The strengths of this study include its prospective nature, real life clinical situation, careful patient evaluation, comprehensive data capture and use of a well validated gastrin radioimmunoassay. Limitations include the single centre study design, the two week cut-off to define PPI use may not have fully accounted for potential prolonged effects of previous PPI consumption on serum gastrin concentrations, lack of repeat gastrin testing to confirm abnormal results, lack of systematically performed contemporaneous clinical blood tests to assess for chronic kidney disease or anti-intrinsic factor/anti-gastric parietal cell antibodies, lack of full Sydney system gastric biopsies to assess preneoplasia, lack of discussion between pathologists to come to a consensus view on preneoplasia cases and incomplete follow up of some patients to exclude subsequent gastrinoma diagnoses.

In conclusion, our results demonstrate that although *H. pylori* infection, gastric preneoplasia and PPI use can all cause elevations of fasting serum gastrin concentrations, the extent of this increase is not predictable and many patients who have risk factors still demonstrate normal fasting serum concentrations of this hormone. Interpretation of fasting serum gastrin concentrations therefore needs to take into account the complete clinical picture and it is not possible to set a cut-off value to identify patients who require further investigation for a gastrinoma. Moreover, as the degree of hypergastrinaemia induced by these factors is highly variable, it is difficult to establish the extent to which gastrin plays a potential role in the development of upper gastrointestinal neoplasms.

## Data Availability Statement

The raw data supporting the conclusions of this article will be made available by the authors, without undue reservation.

## Ethics Statement

Local ethics committee (Liverpool [Adult] Research Ethics Committee REC:08/H1005/37) approval was obtained. The patients/participants provided their written informed consent to participate in this study.

## Author Contributions

ARM and SVM recruited the patients and performed the study. RV-S, ARM, and SVM collected and analysed the data. LT analysed the histopathology. GJD, AV, and DMP designed and supervised the research. RV-S and DMP wrote the paper. All authors approved the final version of the manuscript. The guarantor is DMP.

## Funding

This work was supported by a grant from the National Institute for Health Research (NIHR)-funded Liverpool Biomedical Research Centre for Microbial Disease. R-VS was also supported by a North West Cancer Research student bursary.

## Conflict of Interest

DMP has served as a speaker, a consultant and/or advisory board member for Ipsen, Advanced Accelerator Applications and Mayoly Spindler laboratories and has received research funding to investigate gastric NETs from Trio Medicines Ltd. These funders had no involvement with the current study.

The remaining authors declare that the research was conducted in the absence of any commercial or financial relationships that could be construed as a potential conflict of interest.

## Publisher’s Note

All claims expressed in this article are solely those of the authors and do not necessarily represent those of their affiliated organizations, or those of the publisher, the editors and the reviewers. Any product that may be evaluated in this article, or claim that may be made by its manufacturer, is not guaranteed or endorsed by the publisher.
